# Supplementary figures and images for: Bacterial viability in the built environment of the home
Source: PLoS One. 2023 Nov 8;18(11):e0288092. doi: 10.1371/journal.pone.0288092 (PMC10631670; doi:10.1371/journal.pone.0288092)

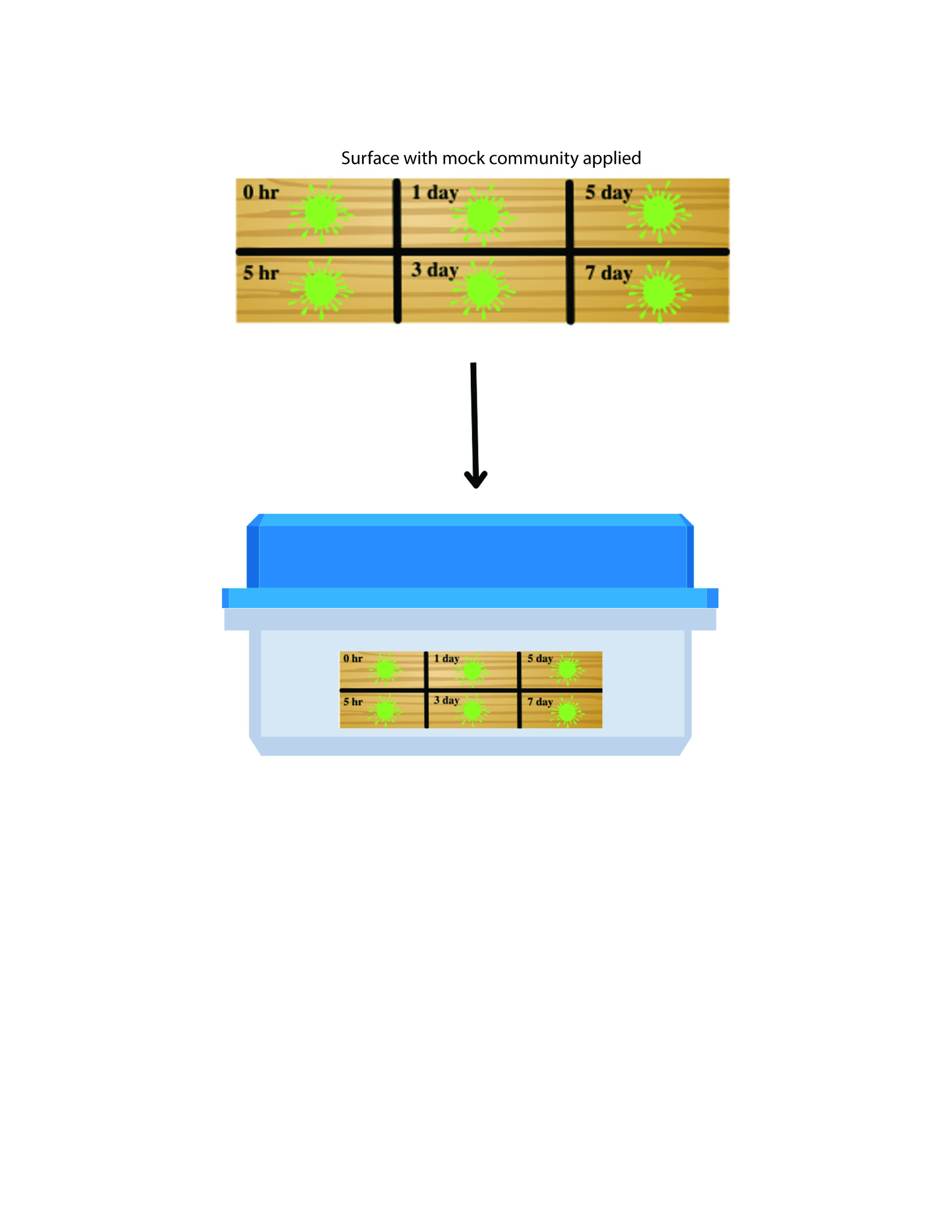

Supplement: S1 Fig — Mock community was plated onto surface and placed into sterile environment. (TIF) [file pone.0288092.s001.tif]

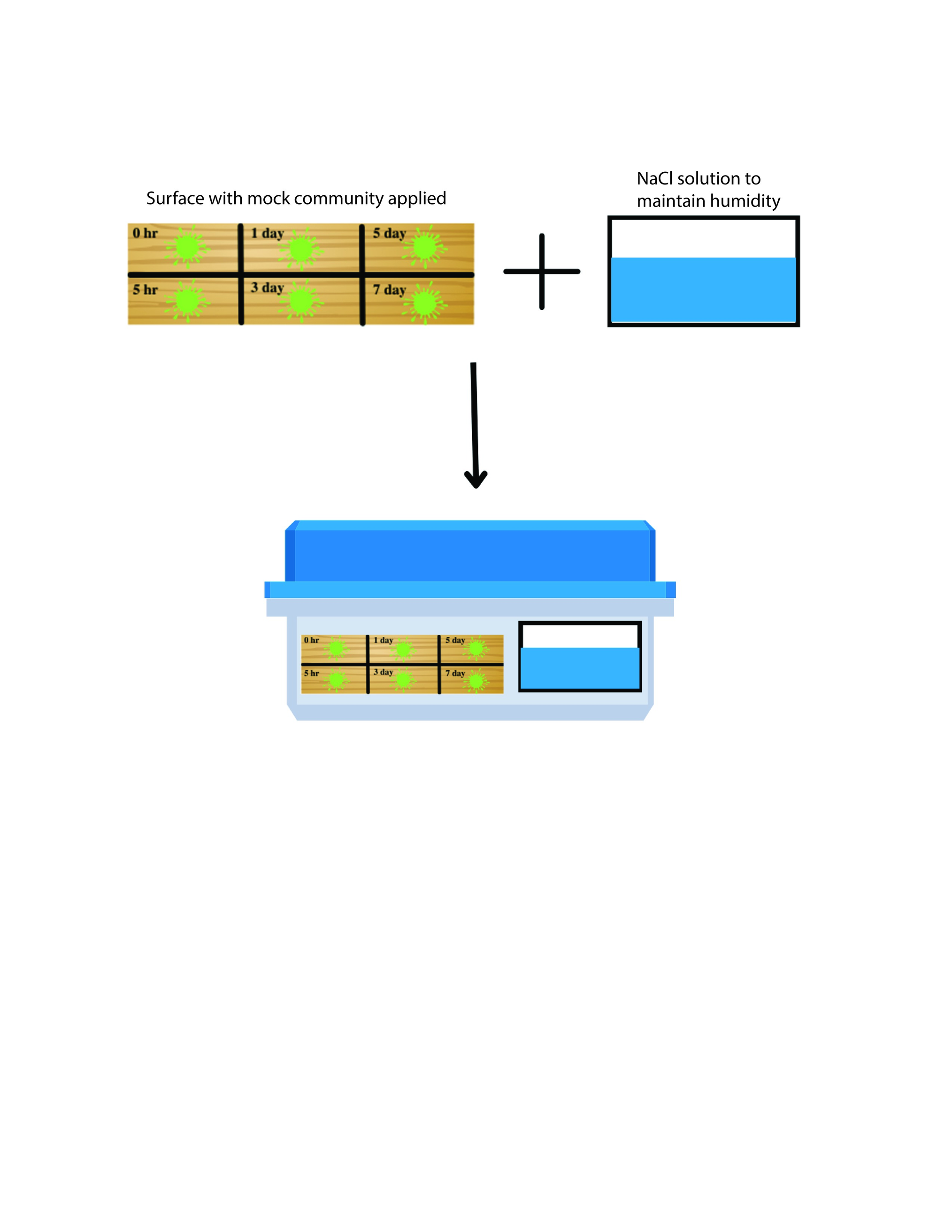

Supplement: S2 Fig — Mock community was plated onto surface. Salt and water solution was created to maintain relative humidity in a sterilized environment. Both were placed into a sterilized environment. (TIF) [file pone.0288092.s002.tif]
