# Supplementary material for: Bacterial viability in the built environment of the home
Source: PLoS One. 2023 Nov 8;18(11):e0288092. doi: 10.1371/journal.pone.0288092 (PMC10631670; doi:10.1371/journal.pone.0288092)
Supplement: S1 Table — A list showing how surfaces were categorized under each location and showing what bacteria species were on each surface. (DOCX) [file pone.0288092.s003.docx]

| **Location** | **Surface** | **Description** |
| --- | --- | --- |
| **Restroom** | Toothbrush | *Rothia dentocariosa* |
|  |  | *Pseudomonas parafulva* |
|  |  | *Streptococcus* sp. |
|  |  | *Kocuria palustris* |
|  |  | *Micrococcus yunnanensis* |
|  | Shower drain | *Delftia* sp. |
|  |  | *Pseudomonas putida* |
| **Kitchen** | Countertop | *Enterobacteriaceae bacterium* |
|  |  | *Acinetobacter baumannii* |
|  |  | *Stenotrophomonas maltophilia* |
|  | Kitchen sink | *Bacillus amyloliquefaciens* |
|  |  | *Acinetobacter calcoaceticus* |
|  |  | *Acinetobacter junii* |
|  | Cutting board | *Bacillus thuringiensis* |
|  |  | *Staphylococcus vitulinus* |
|  |  | *Micrococcus* sp. |
|  |  | *Acinetobacter ursingii* |
|  |  | *Moraxella osloensis* |
|  | Stove knob | *Bacillus aryabhattai* |
|  |  | *Staphylococcus epidermidis* |
|  |  | *Acinetobacter ursingii* |
|  |  | *Staphylococcus pasteuri* |
|  | Faucet | *Acinetobacter ursingii* |
|  | Fridge | *Staphylococcus epidermidis* |
|  |  | *Acinetobacter ursingii* |
|  | Sponge | *Enterobacter* sp. |
|  |  | *Acinetobacter baumannii* |
|  |  | *Stenotrophomonas maltophilia* |
| **Bedroom** | Wall | *Stenotrophomonas maltophilia* |
|  | Ceiling | *Staphylococcus epidermidis* |
|  |  | *Acinetobacter ursingii* |
|  |  | *Stenotrophomonas maltophilia* |
|  | Floor | *Bacillus thuringiensis* |
|  |  | *Staphylococcus epidermidis* |
|  |  | *Acinetobacter ursingii* |
|  | Keyboard | *Staphylococcus aureus* |
|  |  | *Staphylococcus hominis* |
|  |  | *Staphylococcus capitis subsp. Capitis* |
|  | Card | *Staphylococcus aureus* |
|  |  | *Staphylococcus hominis* |
|  |  | *Staphylococcus epidermidis* |
|  | Cellphone | *Staphylococcus hominis* |
|  |  | *Moraxella osloensis* |
|  |  | *Stenotrophomonas pavanii* |
|  |  | *Stenotrophomonas maltophilia* |
|  | Pen | *Stenotrophomonas maltophilia* |
|  | Keys | *Paracoccus yeei* |

**Table S1: Surface samples and their bacteria species**. A list showing how surfaces were categorized under each location and showing what bacteria species were on each surface.
