# Supplementary material for: Bacterial viability in the built environment of the home
Source: PLoS One. 2023 Nov 8;18(11):e0288092. doi: 10.1371/journal.pone.0288092 (PMC10631670; doi:10.1371/journal.pone.0288092)
Supplement: S2 Table — The details for each statistical test performed in this study are included here. (DOCX) [file pone.0288092.s004.docx]

| Figure | Panel | Conditions included in comparison | Statistical test | (Adjusted) P value | Significant |
| --- | --- | --- | --- | --- | --- |
| 3 | A | 4C 60% vs 4C 99% | Student’s T test | 0.9852 | No |
| 3 | A | 22C 60% vs 22C 99% | Student’s T test | 0.0842 | No |
| 3 | A | 37C 60% vs 37C 99% | Student’s T test | 0.3743 | No |
| 3 | B | 4C 60% vs 4C 99% | Student’s T test | 0.1294 | No |
| 3 | B | 22C 60% vs 4C 99% | Student’s T test | 0.2011 | No |
| 3 | B | 37C 60% vs 4C 99% | Student’s T test | 0.0437 | Yes |
| 3 | C | 4C vs 22C | Tukey’s multiple comparisons test | 0.6418 | No |
| 3 | C | 22C vs 37C | Tukey’s multiple comparisons test | 0.3499 | No |
| 3 | C | 4C vs 37C | Tukey’s multiple comparisons test | 0.8577 | No |
| 3 | D | Ambient RH vs 60% RH | Tukey’s multiple comparisons test | 0.8495 | No |
| 3 | D | 60% RH vs 99% RH | Tukey’s multiple comparisons test | 0.3664 | No |
| 3 | D | Ambient RH vs 99% RH | Tukey’s multiple comparisons test | 0.1474 | No |
| 3 | E | 4C vs 22C | Tukey’s multiple comparisons test | 0.3713 | No |
| 3 | E | 22C vs 37C | Tukey’s multiple comparisons test | 0.4331 | No |
| 3 | E | 4C vs 37C | Tukey’s multiple comparisons test | 0.9928 | No |
| 3 | F | Sterile Plastic vs. Sterile Wood | Tukey’s multiple comparisons test | 0.9846 | No |
| 3 | F | Sterile Plastic vs. Sterile Styrofoam | Tukey’s multiple comparisons test | 0.9671 | No |
| 3 | F | Sterile Plastic vs. Sterile Metal | Tukey’s multiple comparisons test | >0.9999 | No |
| 3 | F | Sterile Plastic vs. Sterile Glass | Tukey’s multiple comparisons test | 0.9004 | No |
| 3 | F | Sterile Wood vs. Sterile Styrofoam | Tukey’s multiple comparisons test | 0.7760 | No |
| 3 | F | Sterile Wood vs. Sterile Metal | Tukey’s multiple comparisons test | 0.9782 | No |
| 3 | F | Sterile Wood vs. Sterile Glass | Tukey’s multiple comparisons test | 0.9959 | No |
| 3 | F | Sterile Styrofoam vs. Sterile Metal | Tukey’s multiple comparisons test | 0.9756 | No |
| 3 | F | Sterile Styrofoam vs. Sterile Glass | Tukey’s multiple comparisons test | 0.5630 | No |
| 3 | F | Sterile Metal vs. Sterile Glass | Tukey’s multiple comparisons test | 0.8809 | No |
| 4 | A | High vs. low human interaction | Student’s T test | 0.2178 | No |
| 4 | B | Non sterile vs. sterile | Student’s T test | 0.0296 | Yes |
| 4 | C | Non sterile vs. sterile | Student’s T test | <0.0001 | Yes |

**Table S2: Statistical test information for Figures 3 and 4.** The details for each statistical test performed in this study are included here.
