# Supplementary material for: Bacterial viability in the built environment of the home
Source: PLoS One. 2023 Nov 8;18(11):e0288092. doi: 10.1371/journal.pone.0288092 (PMC10631670; doi:10.1371/journal.pone.0288092)
Supplement: Attachment — Submitted filename: PONE-D-23-07442.docx [file pone.0288092.s006.docx]

This manuscript is well written using the data originating from statistically designed experiments. Built environment microbiome study based on inside houses in different locations is interesting and informative to the reader.

Line 60 remove the words undergraduate research project

Line 255 check repetition of the word ‘that’

Line 468: units give in cms.

*****
