# Supplementary material for: Bacterial viability in the built environment of the home
Source: PLoS One. 2023 Nov 8;18(11):e0288092. doi: 10.1371/journal.pone.0288092 (PMC10631670; doi:10.1371/journal.pone.0288092)
Supplement: Attachment — Submitted filename: Response to reviewers.docx [file pone.0288092.s007.docx]

Thank you for the helpful comments and suggestions. In addition to the changes that are detailed below, we have re-structured the paper so that it is in line with the PLOS One guidelines. The Materials and methods section is now after the Introduction section. Line numbers specified below reflect the updated structure.

Review Comments to the Author

Please use the space provided to explain your answers to the questions above. You may also include additional comments for the author, including concerns about dual publication, research ethics, or publication ethics. (Please upload your review as an attachment if it exceeds 20,000 characters)

**Reviewer #1**: Excellent contribution about daily micro biome environment and can apply for hygiene and health care.

Only minor points

1.title is not specifically site enough or geographical site.

We have revised the title to emphasize that we have examined the built environment of the home.

2. Figure 5 must be improve for more easy and clear for reading and publish.

Thank you for bringing this to our attention. All figures will be re-uploaded to ensure high resolution and publication quality.

**Reviewer #2**: This manuscript investigated the viability of bacteria in the built environment using culture-dependent and culture-independent approach. The content of the manuscript fits well with the aim and scope of the journal and should attract a wide readership. Overall quality of the manuscript is good. The manuscript is well written and clearly explained. Therefore, I recommend a minor revision of this manuscript in its current format. Some concerns are listed below.

**Materials and methods**

In general, this section is well written. However, some parts are lengthy and can be reduced in particular the Sanger sequencing part.

The Sanger sequencing portion of the materials and methods has been edited and unnecessary content has been removed (lines 125-133).

Line 388 For one volume,…. Do the authors mean reaction?

“Volume” has been changed to “reaction” (line 90)

Line 389 DNase

“Dnase” has been changed to “DNase” (line 91)

Line 397-399Please add reference for viability score calculation formula

A citation has been added (line 100). The citation refers to: (22) E. M. Acosta, K. A. Little, B. P. Bratton, X. Mao, A. Payne, D. Devenport, Z. Gitai, bioRxiv, in press, doi:10.1101/2021.08.16.455933. We note that this paper is scheduled to be published at *eLife* within a few weeks, so by the time of final submission of this work we should have that citation available.

Line 405 Why did the authors rely on the isolation of cultivable bacteria on blood agar? The use of only blood agar and short incubation period led to a bias toward pathogenic bacteria and fast growing bacteria. There is likely that the authors will miss out on the slow growing bacterial species in the built environment. Please discuss this point in the discussion section.

The following text has been added to the discussion (line 108):

Blood agar was selected because it is an enriched, non-selective media on which a wide variety of bacterial species can grow, including fastidious microorganisms. To ensure that slow-growing colonies were accounted for, plates were examined under a dissecting microscope that allowed even very small colonies to be visualized.

Line 452 I assumed that the authors used BLAST in NCBI, I strongly recommend the use of EzBioCloud website for a more accurate taxonomic assignment of the bacteria. Please add 16S rRNA gene similarity value from BLAST analysis and length of the 16S rRNA gene in Table S1.

We have included an additional table (Table S3) with the following information from BLAST: query, length of query, query coverage, E-value, and percent identity. Table S3 includes representative sequences for each of the bacterial species listed in Table S1.

Line 461-463Are these 5 bacteria for mocking experiment from culture collection or from your own isolation? Please specify accordingly.

We have clarified this point in the methods section. The following text has been added (lines 144-145):

Species included in the mock community came from single colonies isolated during our environmental sampling.

Line 466How did the authors check sterility of each surface?

Line 467 How did the authors sterilize the box and ccheck sterility of it?

All materials were sterilized using 70% ethanol. Sterility was not assessed following ethanol treatment. Text has been added to line (148) to clarify the method used to sterilize the box.

How many replicates for each time point? Only one experiment or repeated experiment?

Figure 3: Each time-course experiment evaluating temperature, relative humidity, and surface type was performed once. Data shown is the average for timepoints T=4, 24, 72, 120, and 168 hours.

Figure 4: Time-courses with each surface material were performed once. Data shown for each surface type is the average for timepoints T=4, 24, 72, 120, and 168 hours.

Please give detail of surface types under investigated in this section.

The following details regarding the surfaces have been added to the methods (lines 150-153):

Five different surface types were used: polystyrene foam (Antique Ceilings, Inc.), unfinished birch plywood (Coff), stainless steel (K & S Precision Metals), polypropylene plastic (pipette tip boxes, USA Scientific), and glass (microscope slides, Fisher Scientific).

Line 481 70g of what?

NaCl was used to maintain humidity. This point has been clarified in the methods (line 518).

Line 481-482Rewrite this sentence “Salt maintains binds to water and prevents water vapor formation, thereby maintaining a lower RH (28).”

This typo has been corrected. This line (167) now reads “Salt binds to water and prevents water vapor formation, thereby maintaining a lower RH (26).”

**Results**

I recommend the authors rewrite this section as in its current format the results are mixed with discussion.

We appreciate the reviewer’s perspective but respectfully believe that there is not too much interpretation in the results section and that the current results section is structured in such a way as to help the reader understand our findings.

There is no information of the accession number of all 16S rRNA gene sequences generated from this study in public database.

Details regarding the sanger sequencing results, including the nucleotide sequences used for bacterial identification via BLAST, have been included in Table S3.

The resolution of all figures are not good enough. They are blurred except for Figure 1.

Thank you for bringing this to our attention. All figures will be re-uploaded to ensure high resolution.

Why there is A in Figure 1?

“A” has been removed from Figure 1.

There is no Figure2D in figure but it was mentioned in the text.

Thank you for pointing out this error. All mentions of Figure 2D should have referred to Figure 2C and have now been corrected.

Figure S2 what is the object on the right hand side.

The object on the right represents the NaCl solution used to maintain the humidity. Labels have been added to both Figure S1 and Figure S2 for clarification.

Table S1 Please add the length of the 16S rRNA gene, accession number and 16S rRNA gene similarity value from BLAST analysis.

The primers used in this study amplify a 1.5kb region of the 16s rRNA gene (see Methods section, line 125-133). Sanger sequencing produces sequences of 600-1000 nucleotides. These sequences were used for BLAST analysis and have been included in Table S3 along with length of query, query coverage, E-value, and percent identity.

Table S1 delete the word “strain” and the word “sp” must not be italicized.

All occurrences of “strain” have been removed and “sp.” is no longer italicized

Table S2 Column 2 what is “Pannel”? Do the authors mean “panel”?.

“Pannel” has been edited to “Panel”

Table S2 Column 3, Suggest change the word “comparison” to something else eg. conditions or treatments

“Comparison” has been changed to “Conditions included in comparison.”

**Reference section**

Please check carefully for errors eg. no journal name+volume+page number for #10, 18, #34 no volume+page number.

References #10, 18, and 34 (now #31) have been correctly formatted.

**Reviewer #3**: This is very interesting and timely study. Authors have documented the built environment microbiome availability in different locations. Data were analyzed and presented accurately and informative to the readers. It is a well written study based on properly conducted experiments.

Comments from attachment: This manuscript is well written using the data originating from statistically designed experiments. Built environment microbiome study based on inside houses in different locations is interesting and informative to the reader.

Line 60 remove the words undergraduate research project

“undergraduate research project” has been replaced with “study” (line 66)

Line 255 check repetition of the word ‘that’

“That” has been removed from line 367.

Line 468: units give in cms.

Units in cm have been provided in line (154)
